# Dynamics of telomere length in captive Siamese cobra (*Naja kaouthia*) related to age and sex

**DOI:** 10.1002/ece3.5208

**Published:** 2019-04-29

**Authors:** Worapong Singchat, Ekaphan Kraichak, Panupong Tawichasri, Tanapong Tawan, Aorarat Suntronpong, Siwapech Sillapaprayoon, Rattanin Phatcharakullawarawat, Narongrit Muangmai, Sunutcha Suntrarachun, Sudarath Baicharoen, Veerasak Punyapornwithaya, Surin Peyachoknagul, Lawan Chanhome, Kornsorn Srikulnath

**Affiliations:** ^1^ Laboratory of Animal Cytogenetics and Comparative Genomics (ACCG), Department of Genetics Faculty of Science Kasetsart University Bangkok Thailand; ^2^ Department of Botany, Faculty of Science Kasetsart University Bangkok Thailand; ^3^ Queen Saovabha Memorial Institute (QSMI) The Thai Red Cross Society Bangkok Thailand; ^4^ Mildpets Animal Hospital Bangkok Thailand; ^5^ Department of Fishery Biology, Faculty of Fisheries Kasetsart University Bangkok Thailand; ^6^ Bureau of Conservation and Research Zoological Park Organization under the Royal Patronage of His Majesty the King (ZPO) Bangkok Thailand; ^7^ Faculty of Veterinary Medicine Chiang Mai University Chiang Mai Thailand; ^8^ Center for Advanced Studies in Tropical Natural Resources, National Research University‐Kasetsart University (CASTNAR, NRU‐KU), Kasetsart University Bangkok Thailand; ^9^ Center of Excellence on Agricultural Biotechnology (AG‐BIO/PERDO‐CHE) Bangkok Thailand; ^10^ Omics Center for Agriculture, Bioresources, Food and Health Kasetsart University (OmiKU) Bangkok Thailand

**Keywords:** age, hormone, sex, snake, telomere

## Abstract

Telomeres comprise tandem repeated DNA sequences that protect the ends of chromosomes from deterioration or fusion with neighboring chromosomes, and their lengths might vary with sex and age. Here, age‐ and sex‐related telomere lengths in male and female captive Siamese cobras (*Naja kaouthia*) were investigated using quantitative real‐time polymerase chain reaction based on cross‐sectional data. A negative correlation was shown between telomere length and body size in males but not in females. Age‐related sex differences were also recorded. Juvenile female snakes have shorter telomeres relative to males at up to 5 years of age, while body size also rapidly increases during this period. This suggests that an accelerated increase in telomere length of female cobra results from sex hormone stimulation to telomerase activity, reflecting sexually dimorphic phenotypic traits. This might also result from amplification of telomeric repeats on sex chromosomes. By contrast, female Siamese cobras older than 5 years had longer telomeres than males. Diverse sex hormone levels and oxidative stress parameters between sexes may affect telomere length.

## 1 INTRODUCTION

Telomeres comprise tandem repeated DNA sequences and associated proteins which form compound structures to protect the ends of chromosomes from deterioration or fusion with neighboring chromosomes. Longer telomeres are more likely to promote chromosomal stability than shorter ones (Blackburn, 2000). Reduction in telomere length is probably caused by reactive oxygen species (ROS) which contribute to reduction in telomere length in vitro (von Zglinicki, 2002), although evidence for this in vivo is mixed (Boonekamp, Bauch, Mulder, & Verhulst, 2017; Reichert & Stier, 2017). The “end replication problem,” along with downregulation of telomerase, which synthesizes telomeric DNA for telomere ends in somatic cells, leads mainly to the partial loss of telomeres in each DNA replication process (Olovnikov, 1973). This process of gradual telomere length attrition has been linked to aging (Blackburn, 1991). The relationship between telomere shortening and age, commonly found in vertebrates, has attracted increased attention in the context of telomere biology. Telomeres behave as biomarkers of somatic redundancy and represent effects of other biological processes on aging (Boonekamp, Simons, Hemerik, & Verhulst, 2013; Young, 2018). Telomeres are known to shorten with increasing age in humans, some birds, and male garter snakes (Benetos et al., 2001; Bronikowski, 2008; Hausmann et al., 2003; Rollings et al., 2017). By contrast, telomere length does not shorten with age in some long‐lived birds and pythons (Hall et al., 2004; Ujvari & Madsen, 2009). Telomere length is involved in cellular senescence and is, therefore, important (Rodier, Kim, Nijjar, Yaswen, & Campisi, 2005). A decrease in the body's ability to regenerate damaged cells is also related to the reduction in body size and telomere length, implying a relationship between telomere length and life history (Hornsby, 2007).

Sex‐specific telomere dynamics are possibly involved in reproductive strategies and sexual selection, in that different levels of sex hormones may induce telomere shortening, and subsequently initiate cellular senescence, resulting in differences in life span between the sexes (Barrett & Richardson, 2011). Sex determination systems may also affect telomere length and life span. Adult mortality tends to be higher in the heterogametic sex in birds (ZZ/ZW system) and mammals (XX/XY system). Reptiles display considerable diversity in their sex chromosomes with both male and female heterogamety (XX/XY and ZZ/ZW), even within the same taxa (Olmo & Signorino, 2005). However, no complete linkage homology is shown between most mammal/reptile XY and bird/reptile ZW chromosomes (Ezaz, Srikulnath, & Graves, 2017; Singchat et al., 2018). Differences in telomere attrition have been found in mammals and XY reptiles of male heterogamety with age‐related sex disparity. This is very rare in birds and ZW reptiles of female heterogamety, although female‐biased mortality is normal in these taxa. Possibilities of sex differences in life span exist because of other deleterious recessive alleles on both autosome and sex chromosome (Barrett & Richardson, 2011), while dynamics of telomere attrition in female heterogamety have not as yet been comprehensively described. To date, only a few ZZ/ZW ectothermic reptilian species have been investigated and further studies are required to elicit more conclusive evidence. Snakes mostly exhibit genotypic sex determination for ZZ/ZW type (Laopichienpong, Muangmai, et al., 2017; Laopichienpong, Tawichasri, et al., 2017; Tawichasri et al., 2017), and telomere dynamics have been characterized in snake species including water pythons (*Liasis fuscus*), garter snakes (*Thamnophis elegans*), and red‐sided garter snakes (*Thamnophis sirtalis parietalis*) (Bronikowski, 2008; Rollings et al., 2017; Ujvari & Madsen, 2009). The present study makes use of precise individual age records from actively maintained populations of Siamese cobra (*Naja kaouthia*) to improve our understanding of age‐ and sex‐related telomere dynamics in ZZ/ZW species. Siamese cobras that are also ZZ/ZW species are sexually dimorphic in relation to body dimensions (head length and tail length), and males are significantly larger than females when they reach their 2nd or 3rd year and become sexually active during their 3rd or 4th year of life (Chaitae, 2011; Meesook, 2008; Singchat et al., 2018). Most females mate every year before migrating to feeding grounds according to their annual estradiol cycle (Tumkiratiwong, Meesuk, Chanhome, & Aowphol, 2012). Age‐related telomere lengths of Siamese cobras were investigated here in relation to sex‐related differences based on cross‐sectional data. The quantification of relative telomere length (RTL) was performed on the whole blood of 80 Siamese cobras (whose ages ranged from 3 weeks to 11 years) using quantitative real‐time polymerase chain reaction (qPCR) to compare snout–vent length (SVL) with age. Sex‐related differences in telomere dynamics are also discussed.

## 2 MATERIALS AND METHODS

### 2.1 Specimen collection and DNA extraction

Blood samples of 51 male and 29 female Siamese cobras were collected from Queen Saovabha Memorial Institute (QSMI), which supplies healthy snakes for venom and antivenom production (Tumkiratiwong et al., 2012), between late February and late August 2017. All cobras were captive‐bred, ranging from one to three generations in captivity. However, original source of the captive population is unknown. All cobras were released immediately after blood sample collection and maintained in QSMI under the normal conditions throughout their lives (see Table A1 in Appendix A). Each cobra was kept in an area 0.25 × 0.4 m^2^ or 0.4 × 0.6 m^2^ based on body size. Snakes were fed once a week with laboratory mice and chicks (Chanhome, Jintakune, Wilde, & Cox, 2001; Tumkiratiwong et al., 2012). Standing water was also provided in small water dishes placed within the enclosures. All cobras were maintained at a temperature gradient of 27–30°C during the daytime. At night, temperatures fell to around 19–24°C across the enclosure. All cobras were exposed to ambient light cycles. The sex of each individual was identified morphologically by mating observations and sexing probes that searched for the male hemipenes (Laszlo, 1975), and confirmed by a molecular sexing approach using specific codominant and dominant DNA markers based on gametologous genes (Laopichienpong, Muangmai, et al., 2017; Laopichienpong, Tawichasri, et al., 2017; Tawichasri et al., 2017). Animal care and all experimental procedures were approved by the Animal Experiment Committee, Kasetsart University (approval no. ACKU59‐SCI‐034), and conducted according to the Regulations on Animal Experiments at Kasetsart University. The snout–vent lengths (SVL ± 1 mm) of all snakes were measured. Blood samples were collected from the ventral tail vein using a 23‐gauge needle attached to a 2‐ml disposable syringe. Syringes contained 10 mM ethylenediaminetetraacetic acid (EDTA). Whole genomic DNA was extracted following the standard salting‐out protocol as described previously by Supikamolseni et al. (2015) and used as templates for qPCR.

### 2.2 Quantification of telomere length

**Figure 1:**
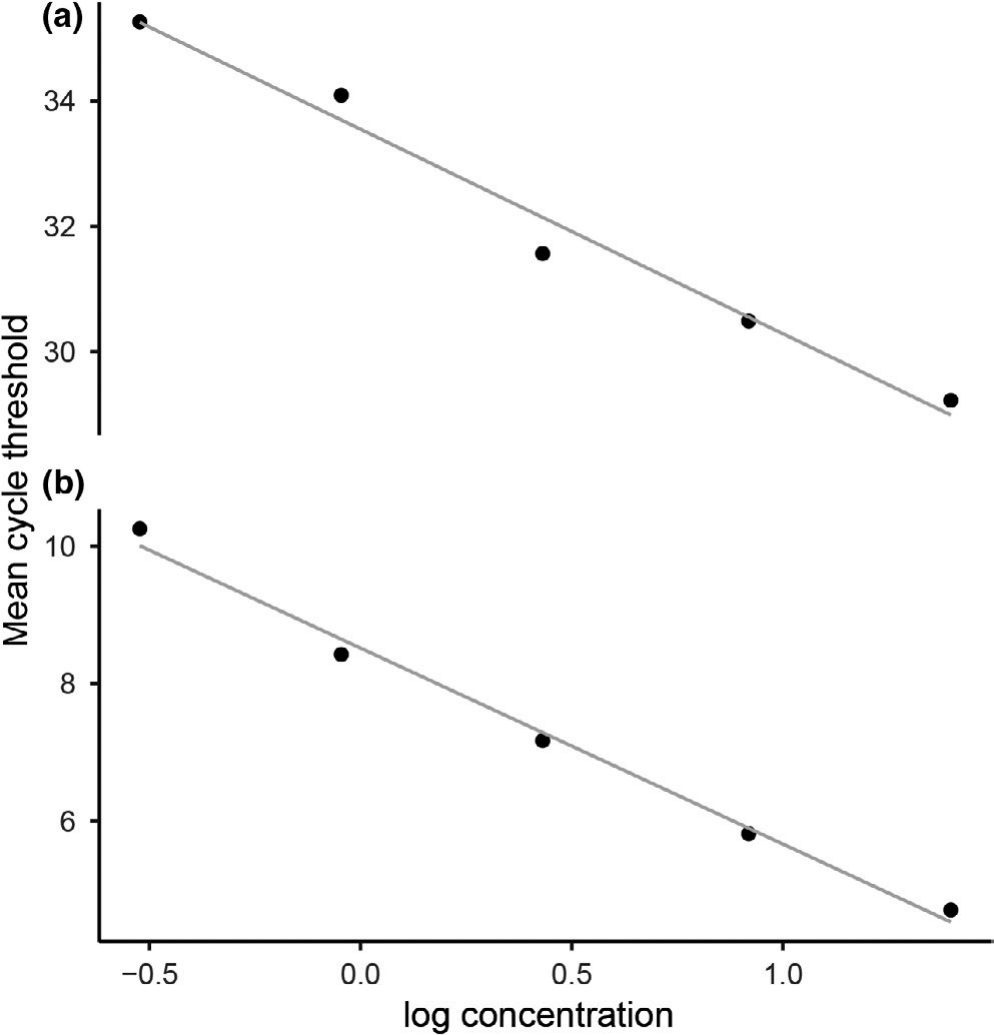
Standard curve determined by GAPDH primers (a) and telomere primers (b). Linear dynamic range from 0.31 to 25.00 ng/µl genomic DNA concentration from Siamese cobra (*Naja kaouthia*) (*n* = 6). Regression lines were calculated as *R* ^2^ = 0.971, *p* < 0.001 for *GAPDH* and *R* ^2^ = 0.987, *p* < 0.001 for telomere length

Telomere length was measured using qPCR with primers Telb1 (5′‐CGGTTTGTTTGGGTTTGGGTTTGGGTTTGGGTTTGGGTT‐3′) and Telb2 (5′‐GGCTTGCCTTACCCTTACCCTTACCCTTACCCTTACCCT‐3′) (Criscuolo et al., 2009). The glyceraldehyde‐3‐phosphate dehydrogenase (*GAPDH*) gene was used as the reference with primer sequences GAPDH‐F (5′‐AACCAGCCAAGTACGATGACAT‐3′) and GAPDH‐R (5′‐CCATCAGCAGCAGCCTTCA‐3′) (Criscuolo et al., 2009). qPCR amplification was performed using 10 μl of 2× KAPA SYBR^®^ FAST qPCR Master Mix (Kapa Biosystems), 0.25 μM primers, and 25 ng of genomic DNA. The PCR conditions were as follows: initial denaturation at 95°C for 10 min, followed by 40 cycles of 95°C for 15 s, 58°C for 15 s, and 72°C for 15 s, with a final extension at 72°C for 5 min. A melt curve over a range of temperatures from 60 to 95°C was created after each run, and the melting profile showed a single peak, indicating no nonspecific product amplification. Telomere length was measured using a Mastercycler ep realplex (Eppendorf AG), and qPCRs were performed in technical triplicate for each specimen template. Control qPCRs were also run in triplicate for each primer set to ensure there was no contamination. Standard curves were produced for both telomere and *GAPDH* using the combined genomic DNA of six randomly selected snakes to ensure consistent rates of amplification over a wide range of DNA concentrations. Threefold serial dilutions were created from a concentration of 25 ng/µl to 0.31 ng/µl with five different concentrations providing a linear dynamic range (Figure 1). According to best practice guidelines (Nolan, Hands, & Bustin, 2006), the reaction was considered in relation to a straight line with *R* ^2^ exceeding 0.971 and 0.989 for *GAPDH* and telomere, respectively, and fitted to the values obtained. Efficiency *E* = −1 + 10^(−1/slope)^ of telomere amplification was 1.10, and the efficiency of *GAPDH* amplification was 1.02. To test intraplate repeatability, intra‐assay coefficient of variation (CV) was measured in triplicate for six samples. Percentage CV for each sample was calculated by searching the standard deviation (*SD*) of each triplicate Ct value of *GAPDH* (S) and telomere (T), respectively, then dividing by the triplicate mean and multiplied by 100. Intra‐assay % CVs should be less than 10 (Hanneman, Cox, Green, & Kang, 2011; Schultheiss & Stanton, 2009). Here, the intra‐assay CV was 2.6266–3.7075 and 6.7238–7.8332 for S and T, respectively. All samples fell within the concentration range generated by the standard curve. The starting concentrations of T and reference gene S were used to calculate the RTL with the calculation of T/S ratio as the copy number of telomeric repeats (Cawthon, 2002; Criscuolo et al., 2009). To test intra‐ and interplate repeatability, eighty cobra samples were run in triplicate on eight different plates to monitor plate‐to‐plate variation. The plate mean for each triplicate value was calculated and subsequently computed to determine the overall mean, *SD*, and % CV. Interassay % CVs of less than 15 are generally acceptable (Hanneman et al., 2011; Schultheiss & Stanton, 2009). In this study, the interassay CV (*n* = 8) was 3.1055, while for intra‐assay CV, it was 1.8027–2.1082 and 4.2070–5.0438 for S and T, respectively.

### 2.3 Statistical analyses

All analyses were performed using R statistical software version 3.4.4 (R Core Team, 2018). Exploratory data analyses were conducted by plotting the relative telomere length (RTL) and snout‐to‐vent length (SVL) against age and sex. Differences between sexes in SVL measurements were determined by Wilcoxon's test. A full model, with the greatest number of RTL variables with age, SVL, and sex as explanatory variables, was constructed and compared with the model without sex (reduced model) using the Shapiro and Levene tests to check the assumptions of normality and variance (data not shown). The model with sex performed significantly better than the model without sex (*F* test, *p* = 0.0002; ΔAIC = 15.64). Furthermore, in the full model, sex by itself was not a significant factor but its interactions with SVL and age were significant (*p* < 0.04; see Table A2 in Appendix A), suggesting that relationships between RTL and other factors were sex‐dependent.

An initial exploratory analysis revealed that the data obtained from males and females were quite different. We then analyzed the sexes separately. To determine snake growth patterns over time, a logistic growth model was fitted to the relationship between SVL and age for each sex using the function “nls” (Fox & Weisberg, 2010). For RTL analysis, a total of six models with age and SVL as explanatory variables were examined using the “lm” function. Adjusted *R* ^2^, Akaike's information criterion (AIC), log‐likelihood, and *p*‐values were retrieved for each model using the “glance” function in the “broom” package (Robinson & Hayes, 2018). A model was considered for selection if the AIC was lower than the competing model by at least four (ΔAIC ≥ 4) (Raftery, 1995). Where two models showed similar statistical values, the one with fewer parameters was selected. Using these results, analyses were performed for each sex separately. A full linear model (RTL ~ age+SVL + age: SVL) for all five reduced models and the quadratic model with age were constructed and compared (Table 1). Analysis of covariance (ANCOVA) was also performed using the “lm” function with sex as the main effect and the age as a covariate, as well as their interaction (sex: age). RTL values were log‐transformed prior to the analysis. The significant interaction terms (*p* < 0.05) indicated that the age‐related change in RTL differed between males and females.

**Table 1:** Linear and quadratic model comparison in male and female Siamese cobra

Model	Adjusted *R* ^2^	*p*‐Value	*df*	logLik	AIC	ΔAIC
Male						
Linear model						
1‐ RTL ~ age	0.4841	<0.001	2	−35.9020	77.8040	2.35
2‐ RTL ~ SVL	0.2141	0.0004	2	−46.6388	99.2777	23.83
3‐ RTL ~ age + SVL	0.4829	<0.001	3	−35.4352	78.8705	3.42
4‐ RTL ~ age:SVL	0.5074	<0.001	2	−34.7260	75.4520	0.00
5‐ RTL ~ age + SVL + age: SVL	0.4869	<0.001	4	−34.7014	79.4029	3.95
Quadratic model						
6‐ RTL ~ age + age^2^	0.4795	<0.001	3	−35.6044	79.2088	3.76
Female						
Linear model						
1‐ RTL ~ age	0.0342	0.1695	2	−28.7124	63.4249	18.49
2‐ RTL ~ SVL	0.0716	0.0867	2	−28.1395	62.2791	17.35
3‐ RTL ~ age + SVL	0.3498	0.0014	3	−22.4292	52.8584	7.92
4‐ RTL ~ age:SVL	0.0228	0.2093	2	−28.8828	63.7656	18.83
5‐ RTL ~ age + SVL + age: SVL	0.5197	0.0001	4	−17.4667	44.9335	0.00
Quadratic model						
6‐ RTL ~ age + age^2^	0.5030	<0.001	3	−18.5326	45.0652	0.13

## 3 RESULTS

**Figure 2:**
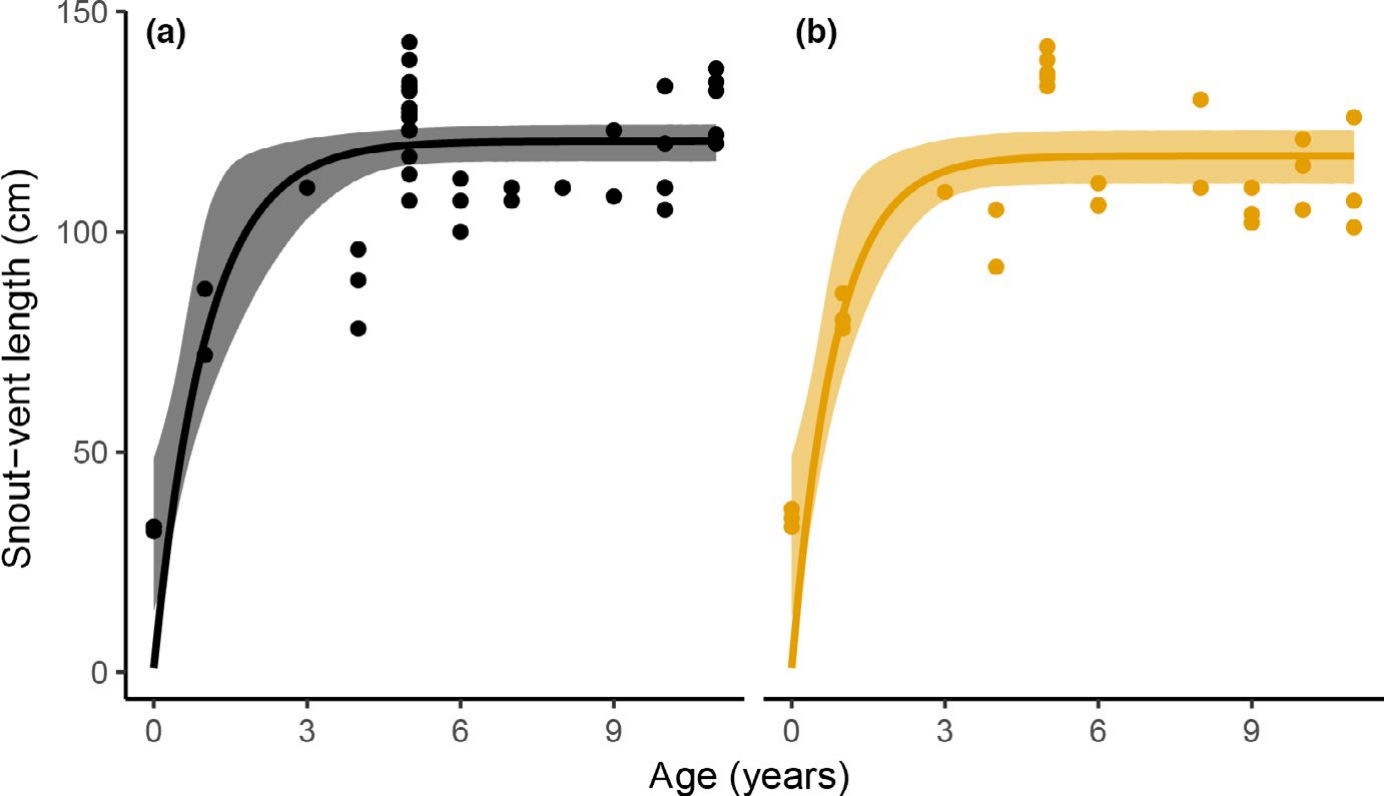
Relationship between age and body size, measured as snout–vent length in male (a) and female (b) Siamese cobra (*Naja kaouthia*) from 0 to 11 years. Solid lines indicate predicted values from the fitted logistic growth functions (male: *R* ^2^ = 0.33, *p* < 0.01; female: *R* ^2^ = 0.35, *p* < 0.01). Shaded areas around the regression lines represent 95% confidence intervals around the predicted values

**Figure A1:**
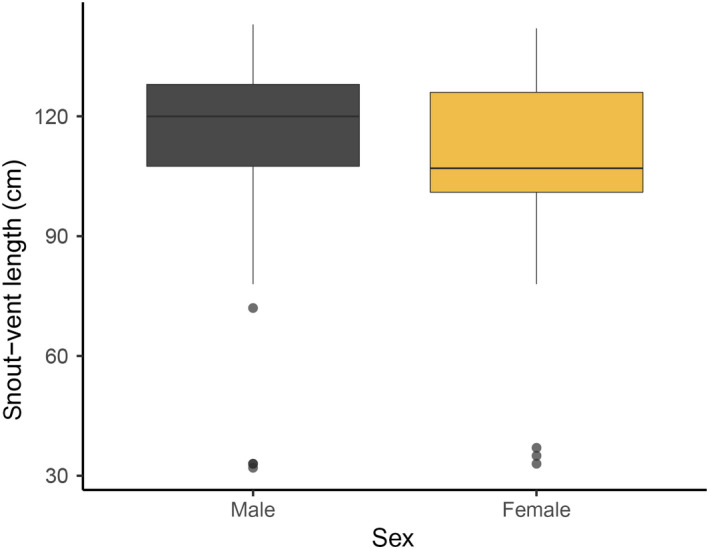
Box plots of snout–vent length (SVL) in relation to sexes of Siamese cobra. No significant difference in SVL between sexes was detected (Wilcoxon's test; *W* = 584, *p* = 0.1205)

**Figure A2:**
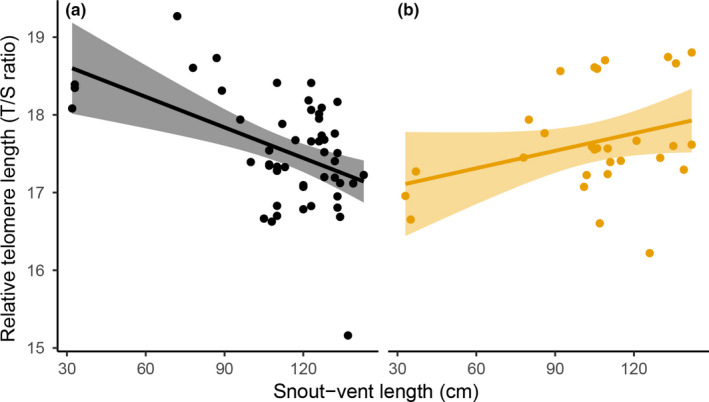
Relationship between relative telomere length (RTL: T/S ratio) and snout–vent length (SVL). Regression lines were calculated separately for (a) males (*R* ^2^ = 0.2141, *p* < 0.01) and (b) females (*R* ^2^ = 0.0716, *p* = 0.08). Shaded areas around the regression lines represent 95% confidence intervals around the predicted values

Estimations of body size based on SVL were conducted to examine the relationships between age and sex for Siamese cobras. Results showed that SVL of both sexes increased with age according to the logistic growth model (male: *R* ^2^ = 0.33, *p* < 0.01; female: *R* ^2^ = 0.35, *p* < 0.01). By fitting the logistic growth model, it was possible to show that SVL reached an asymptote at 120.18 cm in 11‐year‐old males and 111.96 cm in 11‐year‐old females (Figure 2). The Wilcoxon test was performed to determine whether SVL across all ages differed between sexes. No significant differences between sexes (SVL, *W* = 584, *p* = 0.1205; and RTL, *W* = 801, *p* = 0.5415) were found in 0–11‐year‐old snakes (see Figure A1 in Appendix A). Moreover, SVL and RTL were related in males (*R* ^2^ = 0.2141, *p* < 0.01) but not in females (*R* ^2^ = 0.0716, *p* = 0.08; see Figure A2 in Appendix A).

**Figure 3:**
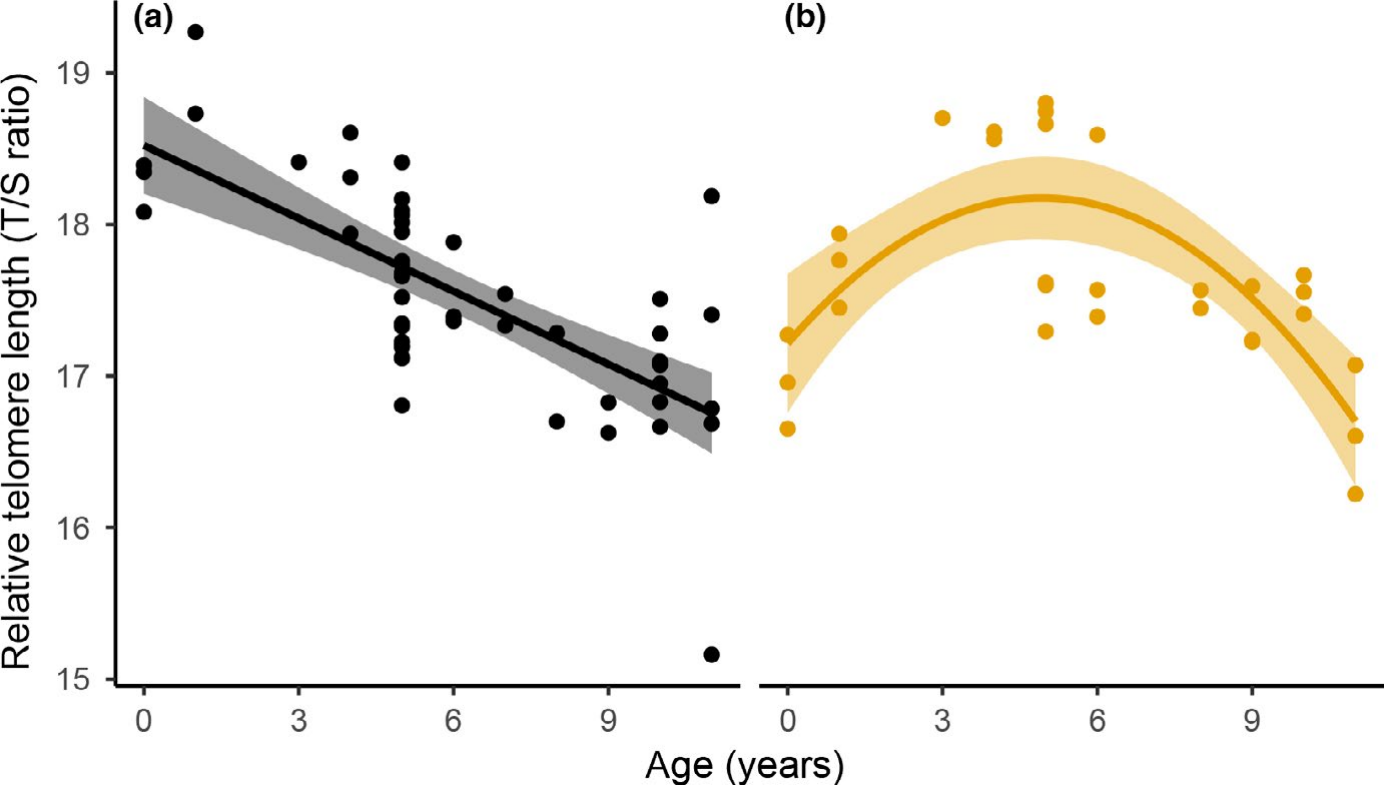
Relationship between age and relative telomere length (RTL: T/S ratio) in male (a) and female (b) Siamese cobra (*Naja kaouthia*) from 0 to 11 years. Solid lines show predicted values from the best‐fitted models as a quadratic model for females and a linear model for males (*R* ^2^ = 0.48, *p* < 0.01). Shaded areas around the regression lines represent 95% confidence intervals around the predicted values

**Figure 4:**
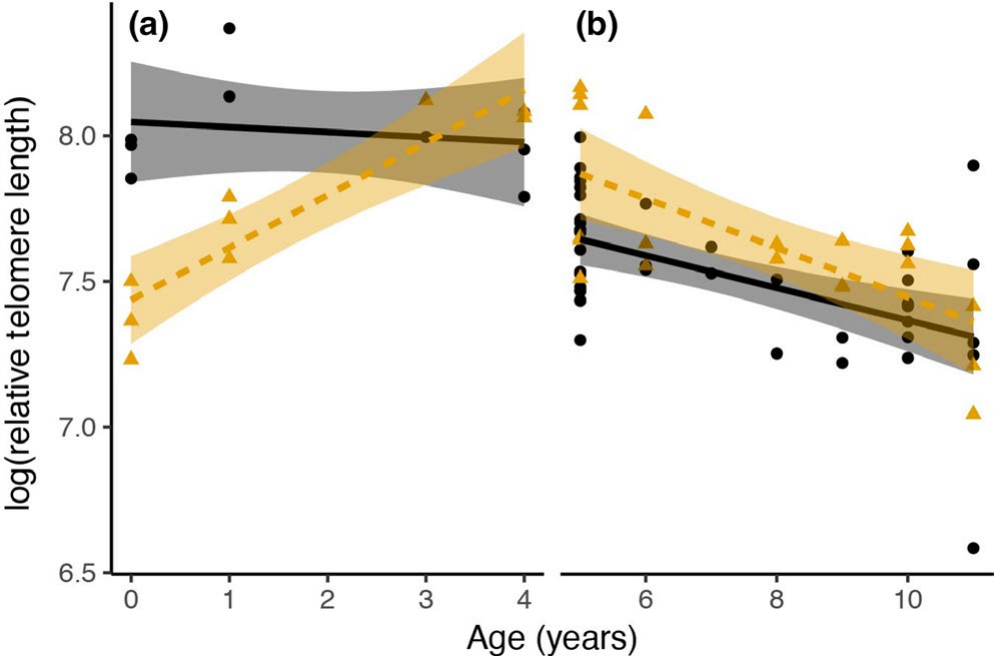
Relationship between age and relative telomere length (RTL) in Siamese cobra (*Naja kaouthia*) from (a) 0 to 5 years and (b) 5 to 11 years. Black circles represent the data from males, and orange triangles represent data from females. Black solid lines and orange dashed lines represent the regression lines for males and females, respectively. Shaded areas around the regression lines represent 95% confidence intervals around the predicted values

Relationships between RTL, age, and body size were examined for each sex using six candidate models (five linear and one quadratic). Linear and quadratic models (between RTL and age) were found to be most suitable and simplest for male and female cobras, respectively (Table 1). The effect sizes, along with 95% confidence intervals (CI), were estimated for the selected models (see Table A3 in Appendix A). However, relationships between age and RTL differed between males and females (Figure 3). In male cobras, RTL decreased linearly with age from 0 to 11 (linear model *R* ^2^ = 0.48, *p* < 0.01), while in females, RTL showed a quadratic relationship with age (quadratic model *R* ^2^ = 0.48, *p* < 0.01), increasing up to 4.91 and then decreasing up to 11 years old. Using this cutoff point of 4.91 years, two separate ANCOVAs were performed for two age ranges, (a) less than 5 years old and (b) greater than 5 years old to determine the relationship between RTL, age, and sex. In the 0–5 years old range, RTL changed significantly with age and differed between sexes (*p*_age_ = 0.002, *p*_sex_ = 0.002; Figure 4a). Interaction between age and sex was also significant (*p*_age:sex_ < 0.01), indicating that age‐related changes in RTL differed between sexes. RTL showed a slight decrease with age in males, but a positive correlation was observed between RTL and age in females (see Table A4 in Appendix A). However, in the 5–11 years old range, RTL decreased linearly with age in both males and females (*p*_age_ < 0.01; Figure 4b). Female telomeres were longer than males (*p*_sex_ = 0.01); however, age‐related changes in RTL were not significantly different between sexes (*p*_age:sex_ = 0.25; see Table A5 in Appendix A). The effect sizes, along with 95% CI, were estimated for models of 0–5‐ and 5–11‐year‐old Siamese cobras (see Tables A6 and A7 in Appendix A).

## 4 DISCUSSION

Potential links between telomere length, age, and sex‐related differences remain poorly understood within ZZ/ZW ectothermic reptiles (Barrett & Richardson, 2011). Body size positively correlated with age for both sexes. This result was at odds with that seen for red‐sided garter snakes, where female but not male body size was related to age (Rollings et al., 2017). By contrast, correlation between body size and telomere length was observed in male but not in female Siamese cobras, suggesting the presence of sex‐dependent telomere dynamics in this lineage.

Contrasting directional correlations between age and telomere length before and after 5 (4.91) years were found in females but not in males, indicating sex bias in telomere maintenance. Juvenile female Siamese cobras have shorter telomeres relative to males at up to five years of age. The dramatic difference in female Siamese cobra telomere lengths before and after the five‐year age point suggests rapid and substantial telomere growth during the first five years of life. Siamese cobras also exhibit an extremely rapid increase in body size during this period (Figure 2), requiring very high cell proliferation and secondary sex characteristics leading to sexually dimorphic phenotypic traits (Klapper, Heidorn, Kühne, Parwaresh, & Krupp, 1998; Madsen & Shine, 2000; Ujvari & Madsen, 2009). Sex hormone estrogens are responsible for the development of female secondary sexual characteristics, and estrogen directly induces telomerase activity (Barrett & Richardson, 2011). This suggests that increase in female telomere length is caused by upregulation of telomerase activity with the sex hormone. An assessment of telomerase activity with sex hormones in the somatic tissues of hatchlings and adult Siamese cobras is required to test this hypothesis. However, a false‐positive result might occur because of the very small sample size for individuals less than 1 year old (see Table A1 in Appendix A). This study only considered cross‐sectional data. Longitudinal data with more samples are necessary to confirm our results. Increase in telomere length across the lower age range has been previously reported in reptiles with frequently repeated measurement of study individuals (Barrett & Richardson, 2011; Olsson, Pauliny, Wapstra, & Blomqvist, 2010; Ujvari & Madsen, 2009). An alternative explanation involves the distribution of telomeric repeats on sex chromosomes. Female sand lizards (*Lacerta agilis*) show heterogametic sex for ZZ/ZW type, and remarkable amplification of the telomeric repeats has been found on the W sex chromosome (Matsubara et al., 2015). A significantly positive correlation between age‐related telomere lengths (range 2–8 years) also points to telomere addition in young sand lizards (Olsson et al., 2010). Specific amplification of telomeric repeats was also found on the W chromosome of Siamese cobra (Singchat et al., 2018), suggesting that co‐opted telomeric DNA amplifications on sex chromosomes drive the increase of telomere length in early life history. However, qPCR cannot be used to differentiate between true and telomeric‐like interstitial telomeric sequences (ITSs) (Augstenová, Mazzoleni, Kratochvíl, & Rovatsos, 2018). This suggests that the longer relative telomere length in female Siamese cobras may simply reflect the inclusion of telomeric repeats in sex‐linked ITSs as seen in qPCR measurements. Further research is necessary to more comprehensively understand the degenerate W chromosome and its links with female fitness in snakes.

Female Siamese cobras older than five years had longer telomeres than males at the same age. Similar age‐related telomere length sex differences were also found in water pythons and other vertebrates (Barrett & Richardson, 2011; Jemielity et al., 2007; Ujvari & Madsen, 2009), indicating a relationship between high mortality and telomere maintenance in somatic cells resulting in shorter life span (Hornsby, 2007; Kirkwood, 1977; Tricola et al., 2018). However, no evidence of a difference in maximum age between sexes in Siamese cobras has been recorded. Additional age and life span information for Siamese cobra are required to confirm this possibility. Contrary to estrogen, testosterone and corticosterone increase susceptibility to oxidative stress in males and might lead to telomeric attrition and cellular senescence (Barrett & Richardson, 2011). This suggests that telomeres were shorter in males than females, possibly resulting from links between sex hormones and parameters of oxidative stress. Increased understanding of oxidative stress levels, hormones, and snake life span is required to elucidate this relationship.

Scenarios of telomere dynamics in females are not consistent across snake species (Bronikowski, 2008; Rollings et al., 2017; Ujvari & Madsen, 2009), suggesting that this phenomenon is not conserved in snakes. Correlations between sex, telomere length, age, and physiological constraints across taxa are necessary to confirm age‐related sex differences in telomere dynamics. Snakes from many diverse environments must also be studied. Shorter telomere length with age might be somewhat obscured by the selection of captive individuals through parental effects. Further research is essential to determine whether telomere shortening in a variety of tissues simply reflects a reduced life span or whether it plays a more causal role with genetic significance.

## CONFLICT OF INTEREST

None declared.

## AUTHOR'S CONTRIBUTION

W.S. and K.S. conceived the ideas, designed the methodology, carried out the laboratory work, and drafted the manuscript; W.S., E.K., V.P., and K.S. participated in data analysis and carried out the statistical analyses. T.P., S.S., and L.C. participated in specimen collection. All authors reviewed the data and gave final approval for publication.

## ETHICAL APPROVAL

Animal care and all experimental procedures were approved by the Animal Experiment Committee, Kasetsart University (approval no. ACKU59‐SCI‐034).

## DATA ACCESSIBILITY

The dataset supporting this article has been uploaded as Appendix. Specimen information including age, sex, body size, and relative telomere length (RTL) of Siamese cobra (*Naja kaouthia*) is shown in Table A1 in Appendix A.

## APPENDIX A

**Table A1:** Body size and relative telomere length (RTL: T/S ratio) of Siamese cobra (*Naja kaouthia*)

Number	Age	Sex	SVL^a^	RTL (T/S ratio)^b^	Sample collection^c^
1	3 weeks	Male	32	71,237,100.28	NK0050560(3)
2	3 weeks	Male	33	92,862,770.87	NK0050560(4)
3	3 weeks	Male	33	97,021,391.11	NK0050560(5)
4	3 weeks	Female	35	17,057,185.00	NK0050560(1)
5	3 weeks	Female	33	23,123,202.91	NK0050560(2)
6	3 weeks	Female	37	31,653,182.89	NK0050560(6)
7	1 year	Male	87	136,397,437.15	NK1070
8	1 year	Male	72	233,662,590.66	NK1071
9	1 year	Female	78	37,867,081.43	NK1060
10	1 year	Female	86	51,823,542.14	NK1066
11	1 year	Female	80	61,686,248.57	NK1067
12	3 years	Male	110	99,077,376.30	NK1051
13	3 years	Female	109	132,484,185.54	NK1047
14	4 years	Male	78	120,176,121.60	NK1040
15	4 years	Male	89	89,623,497.95	NK1030
16	4 years	Male	96	61,742,619.05	NK1035
17	4 years	Female	105	120,972,066.65	NK1044
18	4 years	Female	92	115,285,716.13	NK1037
19	5 years	Male	134	27,272,639.57	NK994
20	5 years	Male	113	33,504,887.08	NK1020
21	5 years	Male	133	77,582,999.71	NK1024
22	5 years	Male	126	62,512,320.00	NK1023
23	5 years	Male	123	69,899,850.04	NK1022
24	5 years	Male	127	50,278,222.39	NK1017
25	5 years	Male	128	47,683,206.44	NK1016
26	5 years	Male	123	46,637,797.65	NK1015
27	5 years	Male	127	71,925,635.18	NK1013
28	5 years	Male	126	46,683,718.06	NK1011
29	5 years	Male	123	98,942,945.93	NK1010
30	5 years	Male	132	29,250,954.89	NK1008
31	5 years	Male	139	27,143,423.47	NK1007
32	5 years	Male	128	29,442,633.60	NK1005
33	5 years	Male	132	51,586,859.57	NK1004
34	5 years	Male	143	30,264,884.51	NK1003
35	5 years	Male	107	34,139,760.41	NK1000
36	5 years	Male	128	40,608,511.95	NK996
37	5 years	Male	117	47,377,532.35	NK991
38	5 years	Male	126	66,493,670.81	NK990
39	5 years	Male	133	19,891,490.94	NK989
40	5 years	Female	139	32,394,675.24	NK993
41	5 years	Female	135	43,979,077.07	NK995
42	5 years	Female	136	127,344,855.59	NK1019
43	5 years	Female	133	138,193,852.87	NK1018
44	5 years	Female	142	146,464,645.84	NK1001
45	5 years	Female	142	44,729,609.07	NK988
46	6 years	Male	107	34,685,970.31	NK986
47	6 years	Male	100	35,749,363.14	NK976
48	6 years	Male	112	58,403,497.37	NK974
49	6 years	Female	111	35,742,879.53	NK985
50	6 years	Female	106	42,654,920.17	NK979
51	6 years	Female	106	118,703,306.38	NK977
52	7 years	Male	107	41,512,020.70	NK962
53	7 years	Male	110	33,666,700.82	NK950
54	8 years	Male	110	17,881,865.55	NK947
55	8 years	Male	110	32,098,524.60	NK949
56	8 years	Female	130	37,736,209.25	NK942
57	8 years	Female	110	42,563,000.95	NK944
58	9 years	Male	123	20,290,489.51	NK879
59	9 years	Male	108	16,605,939.86	NK872
60	9 years	Female	110	30,623,383.31	NK911
61	9 years	Female	102	30,235,399.60	NK881
62	9 years	Female	104	43,582,152.52	NK877
63	10 years	Male	110	20,351,364.96	NK849
64	10 years	Male	120	25,984,872.40	NK795
65	10 years	Male	110	31,929,896.30	NK771
66	10 years	Male	133	22,981,721.29	NK772
67	10 years	Male	133	40,129,633.57	NK773
68	10 years	Male	120	26,555,796.80	NK776
69	10 years	Male	105	17,267,256.34	NK736
70	10 years	Female	105	41,996,011.27	NK750
71	10 years	Female	115	36,333,232.93	NK754
72	10 years	Female	121	47,009,472.16	NK732
73	11 years	Male	134	17,651,804.36	NK725
74	11 years	Male	137	3,837,767.90	NK715
75	11 years	Male	120	19,477,868.84	NK688
76	11 years	Male	132	36,162,888.24	NK682
77	11 years	Male	122	79,093,721.46	NK681
78	11 years	Female	126	11,072,422.73	NK714
79	11 years	Female	107	16,245,542.74	NK711
80	11 years	Female	101	25,971,144.62	NK710

a SVL (snout–vent length) (SVL ± 1 mm).

b RTL (relative telomere length) with T/S calculation giving the copy number of telomeric repeats as the telomere length.

c Number of snake samples deposited in Snake Farm, Queen Saovabha Memorial Institute, The Thai Red Cross Society, Bangkok, Thailand.

**Table A2:** Model comparisons showing the relationship between relative telomere length (RTL), age, SVL, and sex

Variable	*F*‐value	*p*‐Value
Full model: RTL ~ age * SVL * sex: AIC = 2,996.79		
Age	29.57	<0.001
SVL	1.45	0.23
Sex	0.60	0.44
Age:SVL	3.34	0.07
Age:Sex	4.35	0.04*
SVL:Sex	17.16	<0.001*
Age:SVL:Sex	2.94	0.09
Reduced model: RTL ~ age* SVL: AIC = 3,012.44		
Age	23.22	<0.001*
SVL	1.14	0.28
Age:SVL	2.84	0.09

* *p* < 0.05.

**Table A3:** Estimates of effect size and 95% confidence interval (CI) for selected male and female Siamese cobra (*Naja kaouthia*) models

Variable	Effect size	95% CI	*p*‐Value
Best model for male: RTL ~ age			
(Intercept)	18.521	18.204, 18.838	<0.001
Age	−0.16	−0.207, −0.114	<0.001
Best model for female 1: RTL ~ age + SVL + age:SVL			
(Intercept)	15.938	15.124, 16.752	<0.001
Age	0.344	0.044, 0.644	0.03
SVL	0.027	0.017, 0.037	<0.001
Age:SVL	−0.005	−0.008, −0.002	0.004
Best model for female 2: RTL ~ age + age^2^			
(Intercept)	17.216	16.771, 17.66	<0.001
Age	0.391	0.212, 0.569	<0.001
Age^2^	−0.04	−0.055, −0.024	<0.001

**Table A4:** ANCOVA analysis of the relationship between relative telomere length (RTL), age, and sex for 0–5‐year‐old Siamese cobras (*Naja kaouthia*)

Parameter	*df*	Sum of squares	Mean of squares	*F*‐value	*p*‐Value
Age	1	0.32014	0.32014	12.891	0.0029546**
Sex	1	0.33485	0.33485	13.483	0.0025136**
Age:Sex	1	0.47315	0.47315	19.051	0.0006473***
Residuals	14	0.3477	0.02484		

*** *p* < 0.001.

** *p* < 0.01.

* *p* < 0.05.

**Table A5:** ANCOVA analysis of the relationship between relative telomere length (RTL), age, and sex for 5–11‐year‐old Siamese cobras (*Naja kaouthia*)

Parameter	*df*	Sum of squares	Mean of squares	*F*‐value	*p*‐Value
Age	1	1.2996	1.2996	27.9935	<0.0001***
Sex	1	0.30813	0.30813	6.6371	0.01256*
Age:Sex	1	0.06183	0.06183	1.3318	0.25323
Residuals	58	2.69264	0.04642		

*** *p* < 0.001.

** *p* < 0.01.

* *p* < 0.05.

**Table A6:** Estimates of effect size and 95% confidence interval (CI) for 0–5‐year‐old Siamese cobra (*Naja kaouthia*) model

Variable	Effect size	95% CI	*p*‐Value
(Intercept)	7.436	7.288, 7.584	<0.001
Age	0.18	0.113, 0.247	<0.001
Sex	0.611	0.396, 0.825	<0.001
Age:Sex	−0.197	−0.288, −0.107	<0.001

**Table A7:** Estimates of effect size and 95% confidence interval (CI) for 5–11‐year‐old Siamese cobra (*Naja kaouthia*) model

Variable	Effect size	95% CI	*p*‐Value
(Intercept)	8.293	7.956, 8.629	<0.001
Age	−0.085	−0.126, −0.043	<0.001
Sex	−0.369	−0.764, 0.025	0.066
Age:Sex	0.029	−0.021, 0.079	0.253
